# Adjunctive transcranial direct current stimulation to improve swallowing functions in Parkinson's disease

**DOI:** 10.17179/excli2023-6496

**Published:** 2024-01-18

**Authors:** Ali Akbar Dashtelei, Michael A. Nitsche, Mohammad Ali Salehinejad, Amir Hassan Habibi, Jalal Bakhtyiari, Ahmad R. Khatoonabadi

**Affiliations:** 1 Department of Speech Therapy, School of Rehabilitation, Tehran University of Medical Sciences, Tehran, Iran; 2 Department Psychology and Neurosciences, Leibniz Research Centre for Working Environment and Human Factors, IfADo, Dortmund, Germany; 3 German Centre for Mental Health (DZPG), Bochum/Marburg, Germany; 4 Bielefeld University, University Hospital OWL, Protestant Hospital of Bethel Foundation, University Clinic of Psychiatry and Psychotherapy, Bielefeld, Germany

**Keywords:** Parkinson's disease, dysphagia, transcranial Direct Current Stimulation, conventional dysphagia treatment, combination therapy

## Abstract

Swallowing problems are frequent in Parkinson's disease (PD). The aim of this study was to determine the effectiveness of combined transcranial Direct Current Stimulation (tDCS) and Conventional Dysphagia Therapy (CDT) on dysphagia in PD patients. Twenty PD patients with dysphagia were randomized into two groups: combination therapy (anodal tDCS plus CDT) and sham tDCS combined with CDT. Anodal or sham tDCS, bilaterally over the pharyngeal motor cortex, was applied with one mA during the first 20 min (real) or 30 s (sham) of CDT, which was delivered for 30 min. Both groups received twice-daily treatment sessions within two weeks. Swallowing functions were evaluated before, immediately, and one month after the intervention via the Penetration-Aspiration Scale (PAS), and the Swallowing Disorder Questionnaire (SDQ) as the primary outcome measures, and the Dysphagia Handicap Index (DHI) as the secondary outcome measure. The results showed a significant improvement of PAS scores from baseline to post-intervention and baseline to follow-up in both groups without significant differences between groups (t=0.03, p=0.973, and t=1.27, p=0.22 for post-intervention and follow-up time points, respectively). The results showed a significant reduction of SDQ and DHI scores in both groups after the intervention, but the magnitude of the change was significantly larger in the anodal tDCS group at the post-intervention (ta=2.58, pa=0.019 and tb=2.96, pb=0.008) and follow-up (ta=2.65, pa=0.016 and tb=2.97, pb=0.008) time points. This study provides preliminary evidence that bi-hemispheric anodal tDCS combined with CDT enhances swallowing functions in patients with Parkinson's disease more than CDT alone.

## Introduction

Swallowing is a complex sensory-motor function that is bilaterally controlled by a distributed neural network involving not only cortical areas, such as the primary somatosensory and motor cortex, supplementary motor area (SMA), anterior cingulate, and insula, but also subcortical areas, including the pedunculo-pontine tegmental nucleus in the medulla oblongata. Swallowing disorder or dysphagia is common in Parkinson´s disease (PD) and may involve all swallowing phases (Luchesi et al., 2017[19]). The prevalence of dysphagia in PD at different disease stages ranges between 18 % and 100 % (Baijens and Speyer, 2009[4]). Dysphagia results in medical (dehydration, malnutrition, and aspiration pneumonia) and psychosocial (depression and social isolation) complications, decreases the quality of life, and enhances the risk of mortality in PD (Dashtelei et al., 2019[10]).

Therapeutic approaches to treat dysphagia in PD include surgical, pharmacological, and electrophysiological treatments (i.e., surface electrical stimulation), rehabilitation methods such as compensatory strategies, swallowing maneuvers, and behavioral-instrumental approaches such as Expiratory Muscle Strengthening Training (EMST), and Video-Assisted Swallowing Therapy (VAST). However, studies exploring the efficacy of therapeutic approaches to improve swallowing functions have been scarce in PD patients with dysphagia (Monte-Silva et al., 2011[23]).

Over recent years, transcranial direct current stimulation (tDCS), a non-invasive brain stimulation technique, has improved cortical re-organization of swallowing functions in stroke patients (Simons and Hamdy, 2017[32]). It may thus emerge as a complementary approach in swallowing rehabilitation therapy. tDCS modulates the excitability of cerebral target areas at the macro-scale level by subthreshold depolarization (anodal tDCS) or hyperpolarization (cathodal tDCS) of neuronal membrane potentials (Beretta et al., 2020[6]; Tedesco Triccas et al., 2016[37]). Beyond these acute effects, stimulation over some minutes induces neuroplastic after-effects. Anodal tDCS induces long-term potentiation-like plasticity for conventional protocols, while cathodal tDCS generates long-term depression-like excitability diminutions (Stagg and Nitsche, 2011[34]). tDCS-induced plasticity involves the glutamatergic system and is a calcium-dependent process (Nitsche et al., 2003[24]). Presumably via its LTP-like plasticity effects, anodal tDCS has been shown to enhance motor skill learning and to improve motor rehabilitation in corticobulbar-related tasks such as swallowing (Erfmann et al., 2022[12]; Santos Ferreira et al., 2019[28]). Multiple-session anodal tDCS interventions can lead to long-lasting behavioral effects (Stagg and Nitsche, 2011[34]; Tedesco Triccas et al., 2016[37]). Following the general effects of tDCS on motor physiology, Jefferson et al. showed that anodal tDCS increases the excitability of the pharyngeal motor cortex (PMC) in an intensity-dependent manner (Jefferson et al., 2009[14]; Maezawa et al., 2020[20]). Previous studies have moreover shown that anodal stimulation improves symptoms of post-stroke dysphagia (Ahn et al., 2017[1]; Kumar et al., 2011[16]; Pingue et al., 2018[25]; Shigematsu et al., 2013[31]; Suntrup-Krueger et al., 2018[36]; Yang et al., 2012[38]). However, no study has yet evaluated the effect of anodal tDCS on dysphagia in PD. We investigated the effect of multiple-session bilateral anodal tDCS over the PMC, combined with conventional dysphagia therapy (CDT), on swallowing functions in PD patients with dysphagia. We hypothesized that anodal tDCS combined with CDT, due to the induction of long-term plasticity-like effects, would improve PD-related dysphagia more than CDT combined with sham tDCS.

## Materials and Methods

### Study design and randomization 

**Figure 1:**
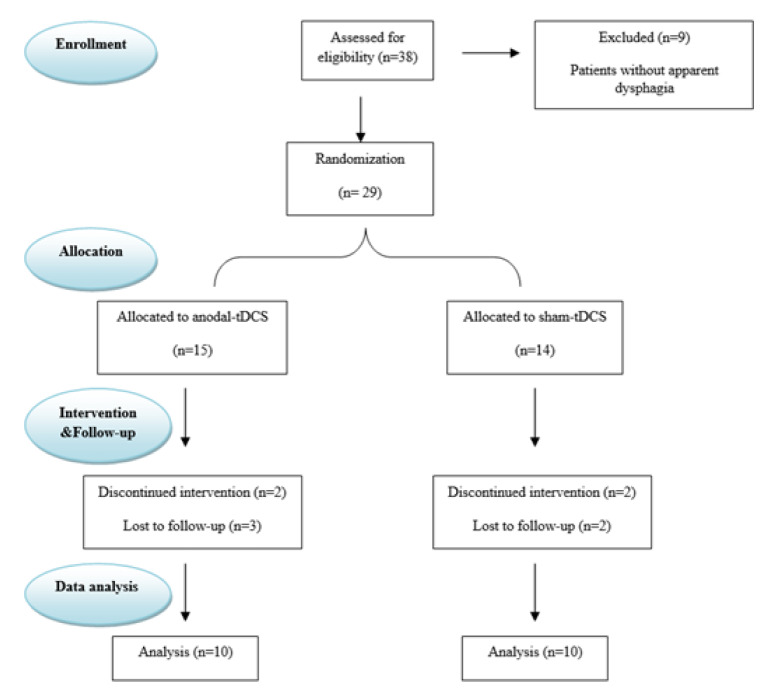
CONSORT diagram. tDCS, transcranial Direct Current Stimulation

We investigated the effects of tDCS on dysphagia improvement in a randomized, single-blind (patients, but not investigators were blinded to the intervention), sham-controlled, parallel-group study. The study was approved by the Research Ethics Committee of the School of Nursing and Midwifery & Rehabilitation - Tehran University of Medical Sciences (IR.TUMS.FNM.REC.1397.179) and was registered at the Iranian Clinical Trial Registry (IRct20190412043248N1). Signed written informed consent was obtained from all participants after reviewing all aspects of the study, including possible benefits, assessment processes, the rehabilitation treatment protocol, risks, and side effects of tDCS. The permuted-block randomization method assigned eligible participants randomly to one of two experimental groups. The study was conducted following the guidelines of the Consolidated Standards of Reporting Trials (CONSORT, Schulz et al., 2010[30]) (Figure 1(Fig. 1)).

### Participants

Parkinsonian patients were recruited through the outpatient neurology clinic at the Rasoul Akram Hospital (Tehran, Iran) between June 2019 and February 2020. Thirty-eight PD patients were assessed for eligibility. Inclusion criteria were diagnosis of Parkinson's disease based on the clinical examination by a neurologist with expertise in PD using the UK brain bank criteria for PD (Daniel and Lees, 1993[9]), absence of other neurological and muscular disease, no presence of metallic implants such as an implanted deep brain stimulation (DBS) device or pacemaker, diagnosis of dysphagia due to PD based on the clinical swallowing examination and Fiberoptic Endoscopic Evaluation of Swallowing (FEES), no history of swallowing therapy, and a Mini-Mental State Examination (MMSE) score larger than 23 at enrollment (Ansari et al., 2010[3]). Exclusion criteria were the presence of any other neurological disorders (such as stroke, seizures, epilepsy, etc.) during the study course, medication with N-methyl-D-aspartate (NMDA) receptor antagonists or Na+ or Ca2+channel blockers, tobacco smoking or being pregnant, a history of alcohol abuse, and use of benzodiazepines or an anticonvulsant.

Nine out of 38 patients were excluded due to a lack of symptoms of dysphagia, according to their clinical swallowing examination and FEES results. The twenty-nine remaining patients were randomly allocated into study groups. Out of 29 patients who commenced the study, nine patients (5 patients from the anodal tDCS group and four patients from the sham tDCS group) were excluded because they failed to complete the study process (due to travel, sickness and family problems five of them failed to participate in the follow-up evaluation, and four others were unable to continue the intervention sessions).

The patients were blinded to the stimulation condition. Therefore, at the beginning of each intervention session, patients in the sham and real stimulation groups were asked whether they felt the onset of stimulation in the area where the electrodes were placed on the scalp. After confirming that all patients perceived the stimulation, the intervention session began.

#### Outcome measures

The PAS and SDQ scores were evaluated as primary outcome measures, and the DHI score as secondary outcome measures. The FEES examined the swallowing function according to the Langmore protocol (Langmore, 2017[18]). These outcome measures were obtained three times: before the first intervention session (baseline), immediately after the last session, and one month after the last session.

### Procedure

#### Assessment protocol

All assessments were conducted in the “ON” state of the patients (approximately one hour after administering 100 mg L-DOPA, or the time of peak concentration of the respective L-DOPA equivalent dosage of a dopamine agonist; the average daily dose of the patients was between 300 and 400 mg L-DOPA equivalents) (Schade et al., 2020[29]). At baseline assessment, demographic data (such as age, gender, disease duration, and medication), clinical characteristics (such as motor disability, cognitive status, and swallowing function), and the outcome measures were evaluated by an experienced speech-language pathologist (SLP) and a neurologist (Table 1(Tab. 1)). The neurologist assessed all patients with the modified Hoehn &Yahr (H-Y) scale. The SLP evaluated swallowing functions via the Penetration-Aspiration Scale (PAS), the Persian version of the Swallowing Disturbance Questionnaire (P-SDQ), and swallowing-related quality of life by the Persian version of the Dysphagia Handicap Index (P-DHI). Both, SDQ and DHI questionnaires are validated in the Persian language (Rajaei et al., 2014[26], Barzegar-Bafrooei et al., 2016[5]).

#### Assessment tools

*a. Fiberoptic endoscopic evaluation of swallowing (FEES)*

The FEES was performed using a CMOS Video Rhino-Laryngoscope device (Karl Storz, Germany) based on the Langmore protocol and was carried out by a trained SLP, using solid, semi-solid, and liquid textures with volumes of 5 and 10 ml (Langmore, 2001[17]). The FEES indicates anatomical structures, functions such as swallowing different consistencies of food and liquid, and the effect of therapeutic interventions such as postural changes. The PAS score was used to quantify the FEES results.

*b. Penetration-Aspiration Scale (PAS)*

The PAS is an 8-point scale used to identify the presence, depth, and response to airway invasion of textures during the FEES. It scores between 1 (material does not enter the airway) and 8 (material enters the airway). Scores from two to five reflect material penetration into the supraglottic space up to the true vocal cords, while scores from six to eight reflect aspiration of material below the true vocal cords (Rosenbek et al., 1996[27]).

*c. Persian version of Swallowing Disturbance Questionnaire (P-SDQ)*

The SDQ is a self-report questionnaire specifically used to assess dysphagia in patients with PD and includes 15 items that examine swallowing problems in the oral (questions 1-5) and pharyngeal (questions 6-15) phases. In the SDQ, fourteen items are rated on a 4-point scale (0-3) (0 for no disturbance and 3 for severe disturbance), and the question "Have you suffered from a respiratory infection (pneumonia, bronchitis) during the past year?" has to be answered with "yes/no" (score 2.5 for yes, and 0.5 for no). The total score of the SDQ ranges between 0.5 and 44.5. A score of 12 or higher is susceptive for dysphagia and requires a more detailed evaluation of swallowing (Rajaei et al., 2014[26]).

*d. Dysphagia Handicap Index (P-DHI)*

The DHI is a self-report questionnaire that includes 25 items subdivided into three subscales: physical (9 items), functional (9 items), and emotional (7 items). Each question has three response options, including never, sometimes, and consistently, scored with 0, 2, and 4, respectively. The total score of this test ranges between 0 and 100. The closer the score is to 100, the lower the quality of life. In addition, each patient evaluates his/her swallowing functions, scoring from 0 (normal) to 7 (severe difficulty) (Barzegar-Bafrooei et al., 2016[5]).

#### Intervention programs

**Figure 2:**
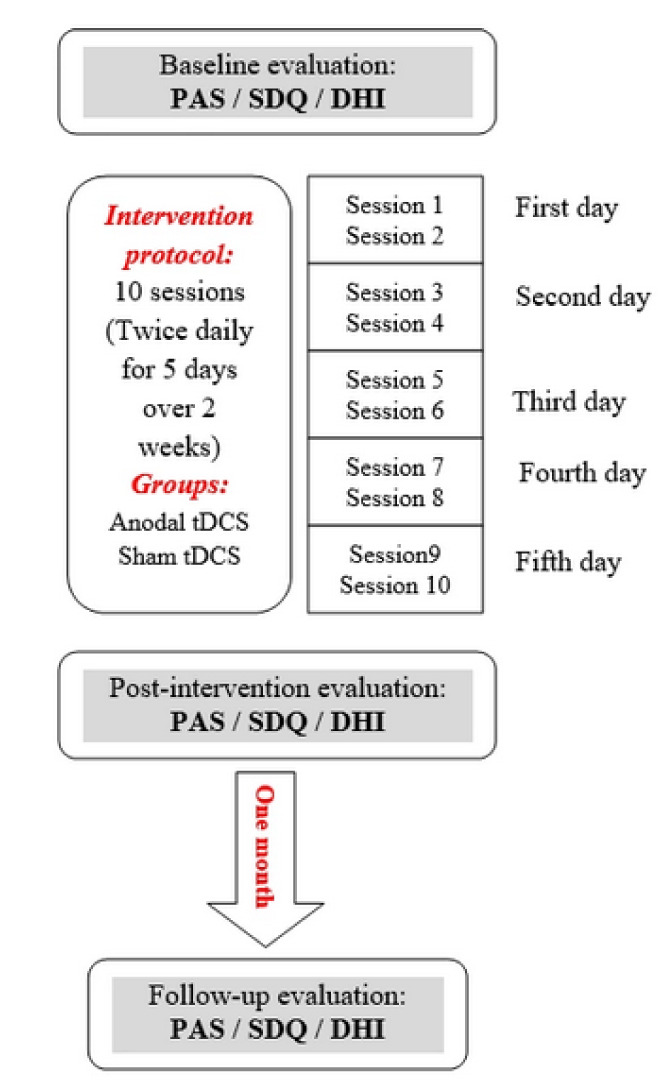
Treatment and evaluation protocol. Anodal tDCS or sham tDCS combined with CDT were simultaneously delivered to all patients for ten 30 min sessions (twice daily with an interval of 10 min for five days over 2 weeks). For the first 20 minutes of each session, the patients received simultaneous anodal tDCS or sham tDCS with CDT, and for the last 10 minutes, CDT only was continued.

Anodal/sham tDCS was applied in conjunction with Conventional Dysphagia Therapy (CDT). All patients received anodal tDCS or sham tDCS combined with CDT simultaneously in ten 30-minute sessions (twice daily with an interval of 10 min for five days over two weeks) (Figure 2(Fig. 2)). Within the first 20 minutes of each session, the patients received simultaneous anodal tDCS or sham tDCS with CDT, and for the last 10 minutes, CDT only was continued.

*a. tDCS protocol*

tDCS was applied with a wireless, battery-driven current stimulator (Starstim, Neuroelectrics, Barcelona, Spain) through two pairs of conductive rubber electrodes covered by saline-soaked (0.9 % NaCl) sponges (size 5 cm x 7 cm = 35 cm^2^). The anodal electrodes were located over the pharyngeal motor cortex (PMC) (C3/C4 according to the 10-20 international electroencephalogram system) (Steinmetz et al., 1989[35]), and the reference electrode was placed above the central supraorbital region (Fpz). Stimulation was conducted with one mA per target electrode (current density= 0.28 A/m^2^) for 20 minutes per session (10 sessions in total, two daily sessions at five days within two weeks), with an interval of 20 min between the two daily sessions. It has been shown that such spaced protocols induce late-phase plasticity and, therefore, relevantly longer after-effects than single daily protocols (Monte-Silva et al., 2013[22]). At the beginning and the end of the stimulation period, the current was gradually ramped up and down over 10 seconds. The patients were sitting on a comfortable chair during the session. The same protocol was applied for sham tDCS, but here the stimulation stopped after 30 seconds. After each session, the patients were asked to report any itching and tingling sensation via the questionnaire introduced by Fertonani and co-workers (2010[13]).

*b. Conventional Dysphagia Treatment (CDT)*

The CDT consists of a structured program based on individual swallowing functions according to a clinical swallowing examination. CDT was provided to all patients and included direct and indirect therapy. The direct therapy consisted of compensatory strategies (such as postural changes and diet modification), and swallowing maneuvers (such as the Mendelsohn maneuver, which includes effortful and supraglottic swallowing). Indirect therapies consisted of Expiratory Muscle Strength Training (EMST), Video-Assisted Swallowing Therapy (VAST), Oral Motor Exercise (OME), dry swallowing, thermal stimulation, tactile stimulation, shaker exercise, and chin tuck against resistance. 

### Statistical analysis

All statistical analyses were conducted using SPSS version 20 (IBM, SPSS, Inc., Chicago, IL). Between-group differences in demographic variables were explored by Chi-square tests, Fisher's exact test for categorical variables, and t-tests for continuous variables. Given the difference between the pre-intervention scores across the groups in some measures, we transformed the raw to standardized scores to eliminate the impact of baseline differences on the outcomes. Standardization was conducted by calculating the quotient of the individual score at a specific time point and the respective individual baseline score. All subsequent analyses were then conducted with the standardized data. To explore the effects of anodal tDCS + CDT on the primary and secondary outcome measures, mixed-model analyses of variance (ANOVA) were conducted on standardized scores of the outcome variables with group (anodal-tDCS vs. sham-tDCS) as the between-subject factor, time (baseline, post-intervention, follow up) as the within-subject factor and standardized scores of PAS, SDQ, and DHI as dependent variables. The normal distribution of the data was evaluated via the Shapiro-Wilk test. The sphericity of the data was explored via the Mauchley test, and the Greenhouse-Geisser correction was applied in case of violation of this condition. In case of significant ANOVA results, pairwise comparisons were conducted with Bonferroni-corrected post-hoc t-tests (two-sided). Cohen's d and eta square (η2) were calculated for effect size calculations. The p-value was set to *p*< 0.05 for all statistical analyses.

## Results

### Data overview

The results of the student's t-tests showed no significant differences between the raw scores of the groups regarding the primary and secondary outcome measures at baseline (*p*<0.05). Therefore, based on the evaluation of the demographic and baseline outcome parameter assessments, the study groups were well-matched, and post-intervention results cannot be attributed to baseline differences. Nevertheless, to exclude a relevant effect of minor baseline differences on the outcome parameters, we standardized post-intervention scores, as outlined above. Descriptive statistics (mean ± standard deviation of outcome measures and demographic information) are presented in Table 1(Tab. 1).

### The impact of tDCS on PAS

**Figure 3:**
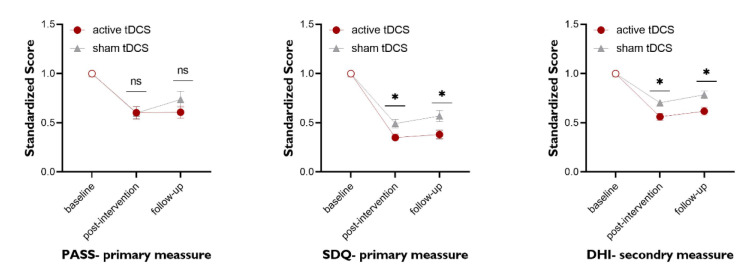
Treatment-dependent changes of swallowing performance in both intervention groups (anodal-tDCS vs sham-tDCS). There was no significant difference between the groups in the respective parameters. All outcome parameters showed a significant improvement in performance after the intervention in both groups. For the primary outcome measures (PASS and SDQ), significant differences between the groups were found both immediately after the intervention and at the follow-up time points only for the SDQ measure, but not for the PASS measure. In the DHI measurement, a significant difference was found between the groups both immediately after the intervention and at the follow-up points. Filled symbols represent significant differences compared to baseline performance. Asterisks (*) represent significant differences between the groups (active vs. apparent) at the respective time point. The error bars show the standard error of the mean (SEM). PAS: Penetration-Aspiration Scale; SDQ: Swallowing Disturbance Questionnaire; DHI, Dysphagia Handicap Index

The results of the mixed model ANOVA showed a significant main effect of time on PAS scores (*F*1.65=48.25, *p*=0.001, *ηp2*=0.72). The main effect of group (*F1*=0.50, *p*=0.485, *ηp2*=0.02), and the time×group interaction (*F1.65*=1.53, *p=*0.233, *ηp2*=0.07) were however not significant. The within-group differences showed a significant reduction of PAS scores from baseline to post-intervention (A) and baseline to follow-up (B) in both active (*tA*=5.125, *p*<0.0001; *tB*=5.061, *p*<0.0001) and sham tDCS groups (*tA*=5.164, *p*<0.0001; *tB*=3.387, *p=*0.004). Nevertheless, the PAS scores in both groups showed no significant difference between the post-intervention (*p=*0.999) and follow-up (*p*=0.299) evaluations. The Bonferroni-corrected critical p-value was 0.0125 (Table 3). Overall, the results show a significant and comparable reduction in the PAS scores of both groups after intervention (Figure 3(Fig. 3)).

### The impact of tDCS on SDQ

The results of the mixed model ANOVA showed a significant main effect of time on SDQ scores (*F*1*.52*=247.95, *p*=0.001, *η*p2=0.93), a significant main effect of group (*F*1=7.67, *p*=0.013, *ηp2*=0.29) and a significant time×group interaction (*F1.52*=5.83, *p*=0.013, *ηp2*=0.24). The within-group comparisons showed a significant reduction of SDQ scores from the baseline to post-intervention (A) and baseline to follow-up (B) in both, active tDCS (*tA*=12.55, *p*<0.0001; *tB*=11.95, *p*<0.0001) and sham tDCS (*tA*=9.808, *p*<0.0001; *tB*=8.321, *p*<0.0001) (Table 3). The reduction of the SDQ score was trend-wise larger for the active than the sham group at the post-intervention (*t*=2.74, *p*=0.024, *Cohen's d*=0.93), and significantly larger after real compared to sham tDCS at the follow-up (*t*=3.63, *p*=0.001, *Cohen's d*=1.2) (Table 2(Tab. 2)). The Bonferroni-corrected critical p-value was 0.008. These results show a significant reduction of the SDQ scores in both groups after intervention, but the magnitude of this change was significantly larger for the active tDCS group (Figure 3(Fig. 3)).

### The impact of tDCS on DHI

The results of the mixed model ANOVA showed significant main effects of time (*F1.74*=129.10, *p*=0.001, *ηp2*=0.87) and group (*F1*=10.27, *p*=0.005, *ηp2*=0.36), and a significant time×group interaction (*F1*.74=6.89, *p*=0.005, *ηp2*=0.27) for the DHI total score. The within-group comparisons showed a significant reduction of the DHI total score from baseline to post-intervention (A) and baseline to follow-up (B) in both, active (*tA*=10.26, *p*<0.0001; *tB*=8.954, *p*<0.0001), and sham tDCS groups (*tA *=6.944, *p*<0.0001; *tB*=5.027, *p*<0.0001) (Table 3(Tab. 3)). The reduction of the total score of the DHI was significantly larger for the active compared to the sham group at the post-intervention (*t=3.32, p=0.004, Cohen's d=1.15*) and follow-up (*t=3.92, p=0.0001, Cohen's d=1.47*) time points (Table 2(Tab. 2)). The Bonferroni-corrected critical p-value was 0.008. These results show a significant reduction of the DHI scores in both groups after the intervention, but its magnitude was larger in the active tDCS group (Figure 3(Fig. 3)).

## Discussion

The results of this single-blinded, sham-controlled, parallel-group study show that anodal tDCS over the bilateral PMC combined with CDT can lead to a long-term stable improvement of swallowing functions, as compared to sham tDCS combined with CDT, and might reduce treatment costs in dysphagic Parkinsonian patients. Furthermore, this intervention was feasible and well tolerated by the study participants.

Few studies have so far investigated the efficacy of swallowing rehabilitation of dysphagia in PD (Dashtelei et al., 2020[11]). Recently, non-invasive brain stimulation has been introduced to increase neural plasticity and thus control and manage PD symptoms via improving rehabilitation success (Broeder et al., 2015[8]). This study investigated tDCS as adjunctive therapy to enhance the efficacy of dysphagia rehabilitation training in PD via induction of long-term potentiation-like plasticity, which has been shown to improve motor learning in health and disease in previous studies (Allman et al., 2016[2]; Michou and Hamdy, 2013[21]; Stagg et al., 2011[33]).

In the present study, the main post-intervention and follow-up results of the PAS score show that swallowing improved relative to baseline performance in both groups, which supports the clinical efficacy of swallowing training. tDCS had no apparent additional effect, except more considerable stability of the improvement, which did not decline at follow-up. For the SDQ score, the other primary outcome measure, and the DHI score, the secondary outcome measure, likewise at post-intervention and follow-up assessments, improved swallowing in both intervention groups was observed. Moreover, between-group assessments of SDQ and DHI scores showed superior performance in the real stimulation group after intervention. This study suggests an adjunctive effect of tDCS, especially for stabilizing the benefits of swallowing therapy. This finding is consistent with those of Khedr and co-workers, who examined the effect of long-term potentiation-like plasticity induction via rTMS on dysphagia in PD (Khedr et al., 2019[15]). 

Generally, the results of previous studies suggested that increasing excitability of the PMC with anodal tDCS combined with CDT, relative to conventional therapy, improves dysphagia symptoms in post-stroke dysphagic patients. Specifically, Kumar and co-workers showed that increasing excitability of the unaffected PMC with anodal tDCS improved dysphagia symptoms in post-stroke dysphagic patients (Kumar et al., 2011[16]). Yang and co-workers applied unilateral anodal tDCS over the PMC of the affected hemisphere in patients with subacute stroke combined with simultaneous CDT. They showed that swallowing functions improved for up to three months after this intervention compared to the sham tDCS group (Yang et al., 2012[38]). Shigematsu and co-workers showed similar effects of anodal tDCS combined with CDT over the ipsilesional PMC in stroke patients (Shigematsu et al., 2013[31]). Ahn and co-workers reported that bi-hemispheric anodal tDCS combined with CDT improved swallowing functions in chronic stroke patients with dysphagia (Ahn et al., 2017[1]). In contrast, Pingue and co-workers showed that anodal tDCS over the damaged hemisphere and cathodal tDCS over the contra-lesional side did not significantly improve post-stroke dysphagia as compared to the sham stimulation group in the early stages of rehabilitation (Pingue et al., 2018[25]). This negative result might be caused by excitability-diminishing stimulation of one PMC in that experimental protocol, which might be dysfunctional for improving swallowing functions because these are bilaterally represented in the motor cortex. In general accordance with the results of previous studies, which combined tDCS with swallowing training, the present study showed positive effects of this intervention in PD, which is not trivial, given the dopaminergic decline in this disease, which has a relevant impact on plasticity, including tDCS effects (Boggio et al., 2006[7]; Monte-Silva et al., 2011[23]). Moreover, we observed long-term stable effects of bilateral twice-daily stimulation and online application during rehabilitation. These effects are more robust than those of some of the studies mentioned above, which might be explained by the fact that the tDCS dose of the present protocol was higher than that applied in most other protocols with respect to the size of the stimulated area, number of sessions, and number of daily interventions. Here, the twice-daily approach, which has been shown to induce late-phase long-term potentiation-like effects at the physiological level, might have contributed (Monte-Silva et al., 2013[22]).

Some limitations of the present study should be taken into account. The disease severity of the study patients was relatively mild to moderate. Although our findings show that bilateral anodal tDCS with simultaneous CDT is useful for swallowing improvement, it is unclear whether this protocol is similarly effective in improving swallowing in more severe stages of PD.

We defined an optimized protocol based on the available data but did not compare it with protocols with different, presumably suboptimal parameters. It would be relevant to test if the protocol conducted in the present study improves intervention efficacy compared to more conventional tDCS interventions.

Given the evaluations performed at follow-up, it would make sense to extend the duration of the follow-up beyond one month to evaluate the actual duration of the after-effects of the intervention and thus further determine clinical suitability.

In this study, only the patients were blinded to the intervention, and the group size was relatively limited, resulting in insufficient power for testing single performance parameters, including PAS, and for detecting relative performance enhancements between interventions in each case.

A longer follow-up would have given relevant information about the stability and, thus, clinical relevance of the obtained effects.

## Conclusion

In summary, the current study revealed that spaced anodal tDCS enhances the efficacy of CDT to improve swallowing problems in PD with respect to the size and stability of the obtained performance improvement. Beyond the swallowing functions, the intervention also improved DHI scores, and thus swallowing-related quality of life in dysphagic PD.

Further investigations to determine optimal tDCS parameters with larger sample sizes and to test effects of this intervention in different disease states of PD would be valuable in future studies.

## Conflict of interest

The authors declare no conflict of interest.

## Figures and Tables

**Table 1:**
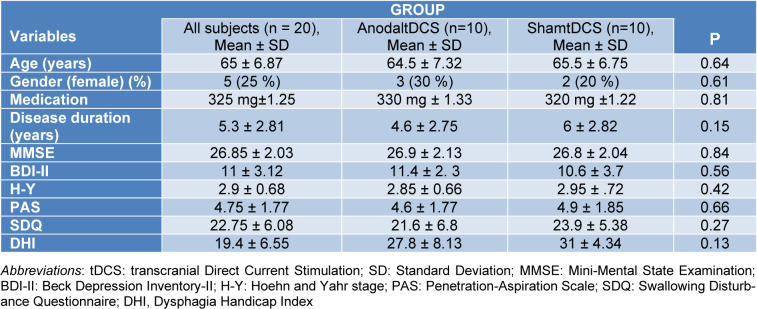
Demographic, clinical characteristics and outcome measures at baseline assessment

**Table 2:**
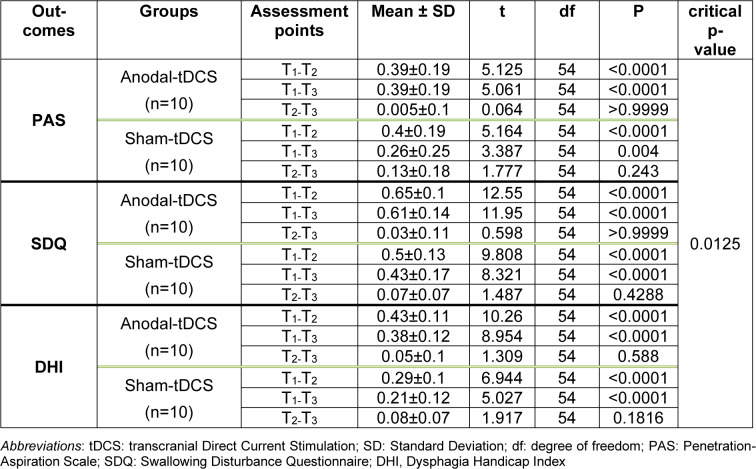
Within-group post hoc t-tests at three assessment points for each group (Bonferroni-adjusted)

**Table 3:**
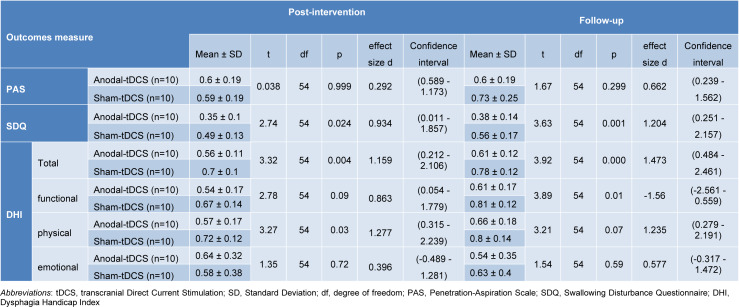
Between-group post-hoc comparisons at the post-intervention and follow-up measures using the Bonferroni correction post-hoc t-tests (two-sided)
